# All-optical control of light on a graphene-on-silicon nitride chip using thermo-optic effect

**DOI:** 10.1038/s41598-017-16989-9

**Published:** 2017-12-06

**Authors:** Ciyuan Qiu, Yuxing Yang, Chao Li, Yifang Wang, Kan Wu, Jianping Chen

**Affiliations:** State Key Laboratory of Advanced Optical Communication Systems and Networks, Department of Electronic Engineering, Shanghai Jiao Tong University, Shanghai, 200240 China

## Abstract

All-optical signal processing avoids the conversion between optical signals and electronic signals and thus has the potential to achieve a power efficient photonic system. Micro-scale all-optical devices for light manipulation are the key components in the all-optical signal processing and have been built on the semiconductor platforms (e.g., silicon and III-V semiconductors). However, the two-photon absorption (TPA) effect and the free-carrier absorption (FCA) effect in these platforms deteriorate the power handling and limit the capability to realize complex functions. Instead, silicon nitride (Si_3_N_4_) provides a possibility to realize all-optical large-scale integrated circuits due to its insulator nature without TPA and FCA. In this work, we investigate the physical dynamics of all-optical control on a graphene-on-Si_3_N_4_ chip based on thermo-optic effect. In the experimental demonstration, a switching response time constant of 253.0 ns at a switching energy of ~50 nJ is obtained with a device dimension of 60 μm × 60 μm, corresponding to a figure of merit (FOM) of 3.0 nJ mm. Detailed coupled-mode theory based analysis on the thermo-optic effect of the device has been performed.

## Introduction

All-optical signal processing avoids the conversion between optical signals and electronic signals and thus a power efficient all-optical system has the potential to replace current electronic systems. A main obstacle preventing the development of all-optical system is the limited capability of large-scale integration. Silicon and III-V semiconductors such as InP and GaAs, are promising platforms for photonic integration^1–4^. Based on these semiconductor platforms, micro-scale all-optical devices for light manipulation have been reported with picosecond response time^1^ or femto-joule energy consumption^2^. However, the finite bandgap of these semiconductor materials introduces the two-photon absorption (TPA) effect and the free-carrier absorption (FCA) effect in the 1550-nm telecom band^3^. These effects increase the light propagation loss and even worse, this loss increases with the injected optical power. The latter results in weak power handling, i.e., the maximum optical power which can be effectively utilized in a photonic chip. This further limits the allowed number of cascaded functional blocks in a semiconductor photonic chip and the capability to realize complex functions.

Silicon nitride (Si_3_N_4_) is a promising candidate material to improve power handling of photonic integrated circuits because its insulator nature guarantees no TPA and FCA at telecommunication wavelength. Recently, various nonlinear applications based on Si_3_N_4_ photonic devices have been demonstrated, including frequency comb generation^5,6^, supercontinuum generation^7^ and so on. Bistability of a graphene-on-Si_3_N_4_ micro-ring resonator (MRR) has also been investigated^8^. However, it is still challenging to effectively manipulate light with Si_3_N_4_. A reported Si_3_N_4_ all-optical switch based on the Kerr nonlinearity (n_2_ = 2.4 × 10^−15^ cm^2^W^−1^) has limited extinction ratio (ER) due to the short interaction length on a chip which makes it difficult for practical switching applications^9^. Thermo-optic effect is another possible solution for all-optical control of light with good extinction ratio^10,11^. But the thermo-optic effect (2.5 × 10^−5^ K^−1^ ^12^) and the thermal conductivity (30 W·m^−1^ K^−1^ ^13^) of Si_3_N_4_ are weaker than those of silicon (1.8 × 10^−4^ K^−1^ and 130 W m^−1^ K^−1^ ^14^), which lead to a low control efficiency and high power consumption. A reported Si_3_N_4_ thermo-optic switch based on MRR has a typical switching time of 5 µs and a switching energy more than 1 µJ^15^. Therefore, a new mechanism is highly desired for the effective all-optical light control in a photonic Si_3_N_4_ device.

Graphene as a two-dimensional (2D) material, has been widely utilized as an efficient absorber for optical modulation^16,17^. Meanwhile, it has an ultra-high thermal conductivity of 5,300 W m^−1^K^−1^ due to the fast phonon transportation in its 2D crystal lattices^18^. Moreover, different from conventional metal heater such as TiN which requires a buffer layer (e.g., SiO_2_ with very low thermal conductivity) to avoid the metal induced huge optical absorption, graphene can directly contact with waveguides since it has a low absorption rate^19^. These two properties result in a highly efficient heat transfer from graphene to waveguides and allow the possibility to significantly enhance the efficiency of the all-optical control on a graphene-on-Si_3_N_4_ integrated platform based on thermo-optic effect.

In this work, we investigate all-optical control of light on graphene-on-Si_3_N_4_ integrated platform. The high thermal conductivity of graphene and the direct contact between graphene and the waveguide effectively compensate the weak thermo-optic effect and thermal conductivity of Si_3_N_4_. The experimental demonstration of all-optical switching with MRR has been realized with a switching time of 253.0 ns, which is 20-fold faster than the abovementioned Si_3_N_4_ thermo-optic switch and even 2-fold faster than a recently reported switch with graphene-on-silicon photonic crystal structure^20^. Detailed modeling and simulation based on coupled-mode theory (CMT) have also been performed to understand the physical dynamics behind the experimental results. Our work reveals the heating and cooling mechanisms of all-optical control of light on an insulator Si_3_N_4_ platform by incorporating graphene and may pave the way to a large-scale integrated all-optical signal processor.

## Results

### Device and principle

**Figure 1:**
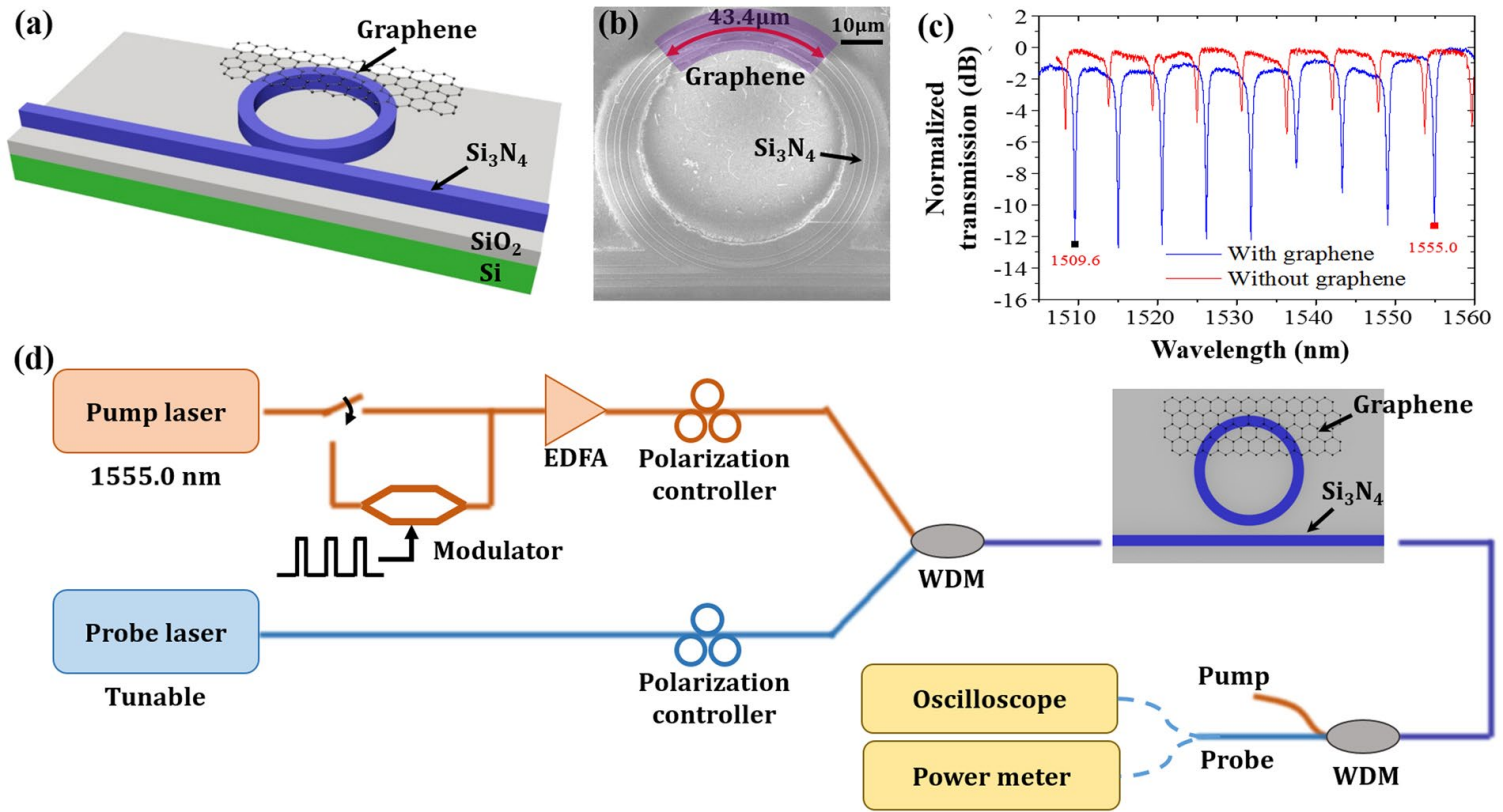
(**a**) The schematic diagram of the all-optical device. (**b**) SEM image of the graphene assisted Si_3_N_4_ all-optical device. (**c**) Transmission spectra of the device before (red) and after (blue) the graphene transfer. (**d**) Experimental setup.

The schematic diagram of the all-optical device is shown in Fig. 1(a). The device consists of a Si_3_N_4_ MRR with a graphene sheet on top. The MRR has a diameter of 60 μm and is side-coupled to a straight waveguide. The gap distance between the MRR and the straight waveguide is set to 100 nm. The waveguides used to construct both the MRR and the straight waveguide have a width of 1.2 µm and a height of 400 nm. Inverse tapers with 1 µm wide tips are integrated for input and output terminals of the waveguides to enhance the coupling between the waveguides and tapered lensed fibers.

The principle of the device is based on the graphene assisted thermo-optic tuning of the resonance wavelength of the MRR. The resonance wavelength of the MRR is given by^21,22^

1 $${\lambda }_{m}=\frac{2\pi {n}_{eff}r}{m}$$

where *r* is the radius of the MRR, *n*_*eff*_ is the effective refractive index of the graphene-on-Si_3_N_4_ hybrid waveguide forming the MRR and *m* is a positive integer known as resonance order. When a pump light at a certain resonance wavelength is injected into the resonator, part of its power is absorbed by the graphene and converted to heat. The heat is then directly transferred to the Si_3_N_4_ waveguide under the graphene. Since Si_3_N_4_ has a positive thermo-optic coefficient of ~2.5∙10^−5^ K^−1^, the resonance wavelength experiences a red shift. If the wavelength of a probe light is set near the resonance wavelength, its transmission will be changed accordingly.

The device was fabricated on a commercial wafer with a 400-nm-thick top Si_3_N_4_ layer and a 5-µm-thick SiO_2_ buried oxide (BOX) layer. The Si_3_N_4_ device was defined by standard electron beam lithography (EBL) and inductive coupling plasma (ICP) etching processes. Chemical vapor deposition (CVD) grown graphene on a copper foil was then wet-transferred on top of the device. After the graphene transfer, the graphene layer was patterned by EBL and oxygen plasma etching processes. Thus only part of the MRR was covered by the graphene sheet after oxygen plasma etching process and the absorption from graphene was reduced. As shown in the scanning electron microscope (SEM) image of the device in Fig. 1(b), the length of the graphene on the MRR is ~43.4 μm.

Figure 1(c) shows transmission spectra of the device with graphene (blue) and without graphene (red). Without graphene, the MRR originally operates at over coupling condition with an extinction ratio of 3~4 dB and a free spectral range (FSR) of ~5.4 nm. After transferring the graphene onto the ring waveguide, the round-trip loss of MRR increases due to the absorption from the graphene. Then the device operates at the critical coupling condition with an ER about 10 dB^22^. The 3-dB bandwidth is 0.52 nm at 1550 nm, corresponding to a Q factor of 2981. Here the graphene induced waveguide propagation loss is estimated to be 113 dB/cm (see Supplement 1). The coupling loss in the experiment is estimated to be ~9 dB/facet.

### Experimental results

The fabricated device is then tested with a pump-probe all-optical control setup, shown in Fig. 1(d). The pump light (control light) generated by a continuous-wave (CW) laser source is either directly injected into an Erbium-doped fiber amplifier (EDFA) for CW pump experiment or modulated by a commercial LiNO_3_ intensity modulator before EDFA for pulsed pump experiment. The wavelength of the pump light beams is set to 1555.0 nm, which matches the resonance wavelength as shown in the spectrum in Fig. 1(c). The probe light (signal light) is generated by a tunable laser source. Pump and probe light beams are combined through a wavelength division multiplexer (WDM) and then coupled to the chip through a tapered lensed fiber. The polarizations of two light beams are controlled by the polarization controllers (PCs) to be quasi-TE. The output light of the chip is coupled out by a second lensed fiber followed by a WDM to separate the pump light and the probe light. The probe light is then characterized by a power meter, a photodetector and an oscilloscope.

**Figure 2:**
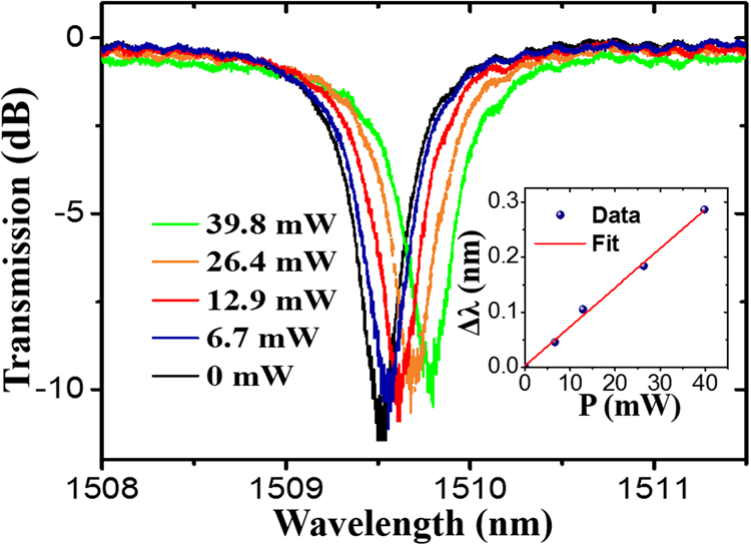
Spectrum response of the device when CW pump is applied. Inset: Resonance wavelength shift with respect to the injected pump power.

The static thermo-optic tuning efficiency of the MRR is first characterized by a CW pump laser source and a tunable laser source as shown in Fig. 1(d). Figure 2 shows the spectrum response of the device near 1509 nm under different pump powers. The corresponding resonance wavelength shift and the phase shift with respect to the pump power is shown in the inset of Fig. 2. The tuning efficiency is 0.0079 nm/mW or 0.00258π/mW. Compared to a similar work based on Si_3_N_4_ MRR without graphene (0.2 nm tuning at 100 mW, corresponding to a tuning efficiency of 0.002 nm/mW)^15^, our efficiency is 4 times higher, which confirms the increased heating efficiency by graphene.

**Figure 3:**
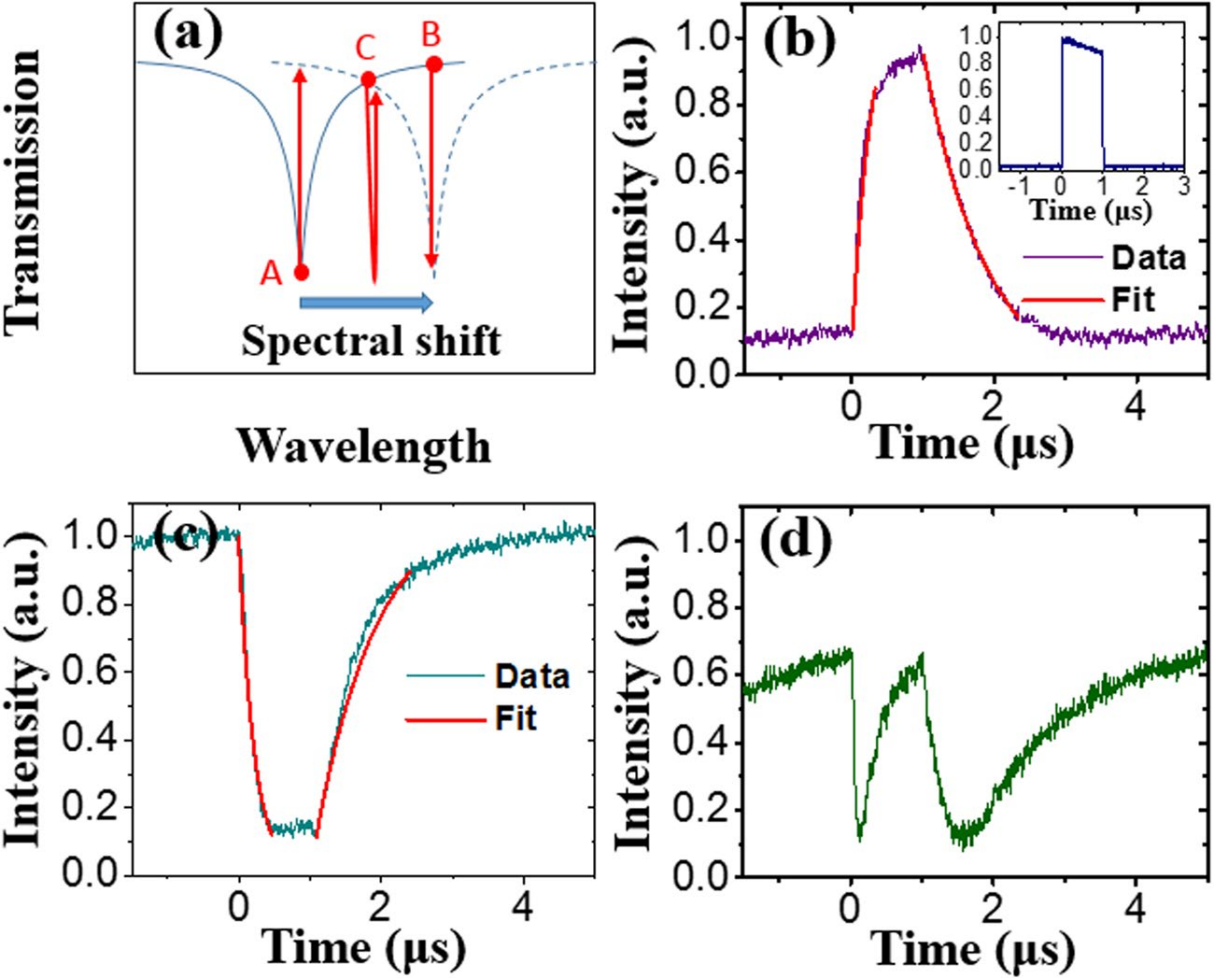
(**a**) Illustration of the bias points of the probe. (**b**) Waveform of pump pulse and probe output biased at point A. (**c,d**) Waveforms of probe output biased at point B and C.

Figure 3 summarizes the dynamic switching properties of the device in the pulsed pump experiment. Here the pump pulse train has a repetition rate of 200 kHz, a pulse width of 1 µs, an injected average power of 40 mW and a duty cycle of 20%, corresponding to a peak power of 200 mW. Figure 3(a) indicates the operation points for different probe outputs. When the probe wavelength is set at point A (1509.56 nm), a pump induced spectral shift results in the generation of bright output pulse of the probe light as shown in Fig. 3(b). It can be observed that the pump pulse induces a switch from ‘off” state (low transmission) to “on” state (high transmission) of the probe. By fitting the rising and falling edges of the waveform with exponential decay functions of 1 − exp(−*t/τ*_*r*_) and exp(−*t/τ*_*f*_), respectively, the time constants are estimated to be *τ*_*r*_ = 253.0 ns and *τ*_*f*_ = 888.3 ns. The rising edge is determined by the heating process from the injected pump power and the heat dissipation to the air and substrate whereas the falling edge is only determined by the cooling process from heat dissipation. When the wavelength is biased at point B of 1510.1 nm, a pump pulse induces a switch from “on” state (high transmission) to “off” state (low transmission) of the probe as shown in Fig. 3(c). The time constants of the falling and rising edges are *τ*_*f*_ = 219.6 ns and *τ*_*r*_ = 860 ns, respectively. In the heating process, the falling edge of the dark pulse is slightly faster than the rising edge of a bright pulse in Fig. 3(b), which is probably due to the slight increased peak power of the pump pulse from the change of the coupling condition between the fiber and waveguide. This phenomenon can also be found in the supplementary material. In the cooling process, the trailing edges of both bright and dark pulses have similar time constants, which means the cooling process is only dependent on the heat dissipation of the device itself. In Fig. 3(b) and (c), both outputs show an ER about 10 dB which is consistent with the ER measured in the transmission spectrum near 1509.56 nm as shown in Fig. 1(c). If the wavelength is biased at point C of 1509.8 nm in Fig. 3(a), the wavelength sweeps through the notch twice when a pump pulse is applied, which results in a transmission change of “on-off-on-off-on” as shown in Fig. 3(d).

### CMT based Simulation

**Figure 4:**
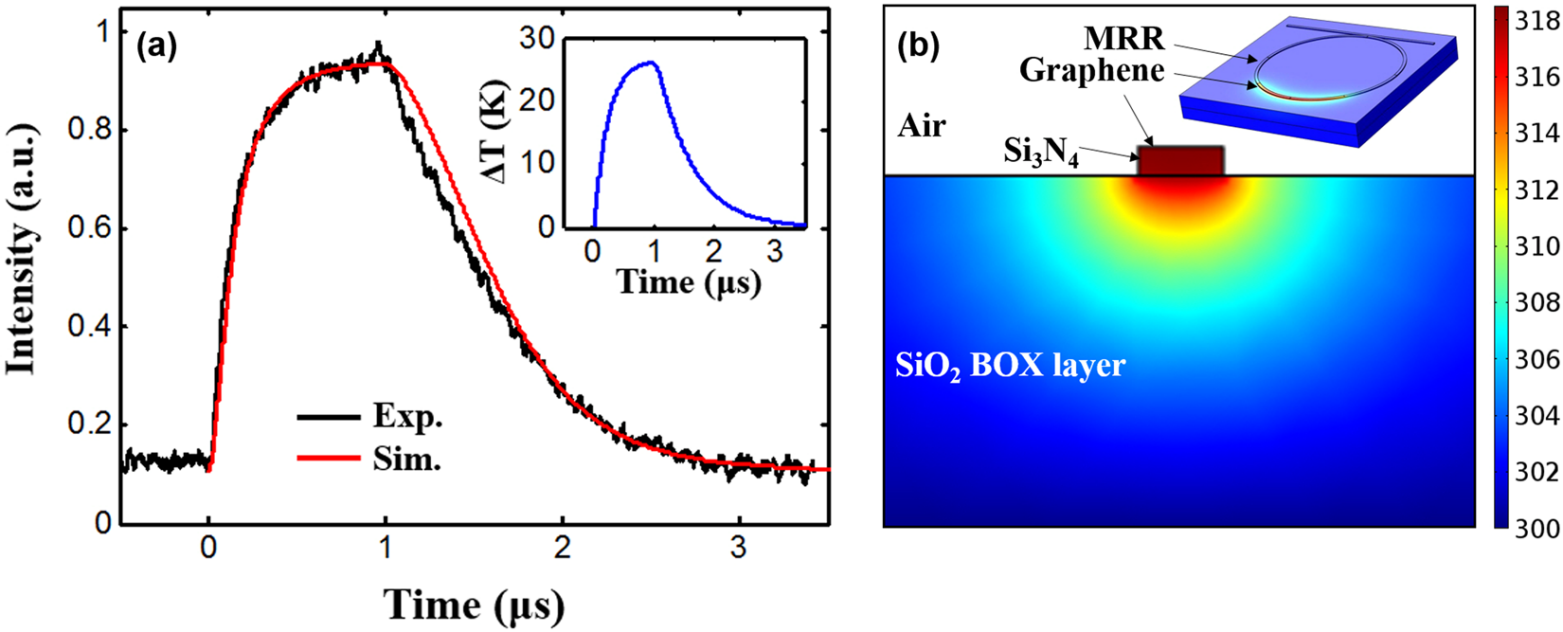
(**a**) Experimental and simulation results of output probe pulse. Inset: average temperature change. (**b**) 2D temperature distribution of the cross section of the graphene covered micro-ring waveguide at 10 mW pump power. Inset: 3D temperature distribution of the device.

Heating process is the key process which determines the device performance. In the heating process, the pump light induces the resonance frequency shift Δ*ω* in the experiment and then the probe signal light is modulated. To clearly understand the dynamic process of the pump light induced switching, the nonlinear time-domain coupled-mode theory has been applied to perform the simulation. The behavior of pump light and the device are described by the following equations^8,23^

2 $$\frac{da}{dt}=(j({\omega }_{0}+{\rm{\Delta }}\omega -\omega )-\frac{1}{{\tau }_{0}}-\frac{1}{{\tau }_{e}})a+\kappa \sqrt{{P}_{in}}$$

3 $$\frac{d{\rm{\Delta }}T}{dt}=\frac{{R}_{th}}{{\tau }_{linear}{\tau }_{th}}{|a|}^{2}-\frac{{\rm{\Delta }}T}{{\tau }_{th}}$$

4 $$\frac{{\rm{\Delta }}\omega }{{\omega }_{0}}=\frac{{\rm{\Delta }}n}{{n}_{g}}=\frac{TOC\cdot {\rm{\Delta }}T}{{n}_{g}}$$

where *ω* is the frequency of the input pump light, *ω*_0_ is the resonant frequency of the cold cavity and Δ*ω* is the time-dependent cavity resonance shift due to the thermo-optic effect. In the experiment, *ω* is equal to *ω*_0_. *a* represents the amplitude for the resonant mode (at the pump light frequency) with the unit of J^−1/2^. 1/*τ*_0_ and 1/*τ*_*e*_ are the decay rates due to the cavity loss and the waveguide-resonator coupling, respectively. *κ* is the coupling coefficient between the waveguide and the resonator. 1/*τ*_*e*_ and *κ* are related to each other by |*κ*|^2^ = 2/*τ*_*e*_. *P*_*in*_ is the pump power in the input waveguide. Δ*T* is the temperature change of the graphene-on-Si_3_N_4_ hybrid waveguide. It should be noted that here Δ*T* represents the average temperature change of the whole micro-ring waveguide because the actual heat distribution is not uniform along the micro-ring waveguide (see the inset of Fig. 4(b)). This treatment is reasonable because the cavity resonance shift and the average index change both can be contributed to the average effect of the temperature change via thermo-optic effect. *R*_*th*_ is the thermal resistor^8^. 1/*τ*_*linear*_ is the linear power absorption rate in the cavity and 1/*τ*_*th*_ is the thermal decay rate. TOC is the thermo-optic coefficient of Si_3_N_4_ which is set to 2.5∙10^−5^ K^−1^. Δ*n* is the time dependent average index change and *n*_*g*_ (=2.20) is the group index of the waveguide. By fitting the transmission spectra of the device (see supplement 1 for more details), we obtain *τ*_*e*_ = 6.22 ps (unchanged with or without graphene), *τ*_0_ = 11.16 ps (with graphene), *τ*_0_′ = 23.23 ps (without graphene) and *τ*_*linear*_ = 10.73 ps. And |*κ*|^2^ = 2/*τ*_*e*_ = 3.21 × 10^11^ s^−1^.

For the resonant mode at the frequency of the probe light, the behavior of the probe light can be similarly described by the following equations^8,23^

5 $$\frac{da^{\prime} }{dt}=(j({\omega }_{0}^{\prime} +{\rm{\Delta }}\omega -\omega ^{\prime} )-\frac{1}{{\tau }_{0}}-\frac{1}{{\tau }_{e}})a^{\prime} +\kappa \sqrt{{P}_{in}^{\prime} }$$

6 $${S}_{out}^{\prime} \,=\sqrt{{P}_{in}^{\prime} }-{\kappa }^{\ast }a^{\prime} $$

where *ω*′ is the frequency of the input CW probe light, *ω*_0_′ is the resonant frequency of the cold cavity for the probe light. *a*′ is the resonant mode amplitude. *P*_*in*_′ is the CW probe light in the input waveguide. *S*_*out*_′ denotes the amplitude of the output probe wave whose squared magnitude is equal to the power of output probe wave. Note that, there is no mutual coupling between the two resonance modes due to different frequencies.

By using equations (1)–(6), the dynamic process can be simulated. Here the physical parameters used in the simulation are listed in Table 1. As shown in Fig. 4(a), it can be observed that simulation result on the pulse profile match the experimental result. The time dependent average temperature change is also plotted in the inset of Fig. 4(a). The maximum temperature change is ~26 K corresponding to a maximum wavelength shift of ~0.45 nm.

**Table 1:** Estimated physical parameters for CMT based simulation.

Parameter	Symbol	Value
Group refractive index	*n*_*g*_	2.20
Decay time due to the cavity loss with graphene	*τ*_0_	11.16 ps
Decay time due to the cavity loss without graphene	*τ*_0_′	23.23 ps
Decay time due to the coupler	*τ*_*e*_	6.22 ps
Coupling strength	|*κ*|^2^	3.21∙10^11^ s^−1^
Decay time due to the linear absorption	*τ*_*linear*_	10.73 ps
Thermal decay time	*τ*_*th*_	603 ns
Thermal resistor	*R*_*th*_	1.37 K/mW

A quantitative analysis on the temperature change is also performed based on the simulation of 2D cross section of the graphene covered micro-ring waveguide. The calculated temperature distribution is shown in Fig. 4(b) at an injected pump power of 10 mW. The pump power is set low and thus the spectral shift of the MRR can be neglected in the following analysis. The thermal conductivities of graphene, Si_3_N_4_ and SiO_2_ are 2000, 29 and 1.38 W/m∙K, respectively^8,24^. The heat convection coefficient for air is 5 W/m^2^∙K^24^. The graphene thickness is 0.5 nm. It can be found that the maximum temperature change is Δ*T*_*max*_ = 18.4 K. Then the average temperature change Δ*T* can be roughly estimated by considering the ratio between the length of graphene layer (43.4 μm) and the length of the micro-ring (π∙60 μm = 188.5 μm), i.e., Δ*T* = Δ*T*_*max*_∙43.4/188.5 = 4.23 K. The corresponding control efficiency is Δ*T*/10 mW = 0.423 K/mW. When the pump power is increased to 200 mW, the ideal temperature change should be 0.423∙200 K = 84.6 K. However, the actual average temperature change obtained by the above CMT based analysis is ~26 K. This is because in the dynamic operation with a pulsed pump, the absorption of the high pump power (200 mW) causes the shift of the MRR resonance wavelength and the actual absorbed pump power decreases. As a result, the control efficiency also decreases. The temperature of the graphene can be estimated as 26 K/43.4∙188.5 = 113 K.

A 3D temperature at 10 mW pump power is also simulated for illustration and shown in the inset of Fig. 4(b). The graphene thickness in the 3D simulation is set to be 10 nm because the memory cost is too huge to conduct the simulation if the graphene thickness is set to the actual value of 0.5 nm.

## Discussion

To further clarify the performance of an all-optical device on graphene-on-Si_3_N_4_ integrated platform using thermo-optic effect, a comparison between our work and reported similar works with insulator materials (e.g., Si_3_N_4_ and SiO_2_) is provided in Table 2. Here two types of figure of merit are defined. FOM1 is defined as the product of minimum pump energy to switch the state and the typical device length with the units of nJ∙mm. FOM2 is defined as the product of pump power (peak power) to switch the state and the typical device length with the units of mW∙mm. A smaller FOM value represents better device performance on the power consumption and compactness. It can be noticed that all-optical switching based on thermo-optic effect typically requires high pulse energy and has large FOM1s whereas the switching based on Kerr nonlinearity typically requires high peak power and has large FOM2s. By using graphene as a bridge for efficient heat transfer, the lowest FOM1 and second lowest FOM2 has been achieved in our device. Moreover, the demonstrated all-optical device operates in single mode and exhibits a good extinction ratio of 10 dB.

**Table 2:** Comparison of reported works of all optical switching in insulator materials.

Material	Principle	Integration	Operation mode	Switching time	Pump wavelength	Extinction ratio	FOM1 (nJ∙mm)	FOM2 (mW∙mm)	Ref.
Silica fiber	Kerr nonlinearity	No	Multi-mode	400 fs	1030 nm	2.8 dB	1710	>10^9^	^25^
Silica fiber w/ graphene	Thermo-optic effect	No	Single mode	4 ms	980 nm	20 dB	2.2∙10^5a^	55^a^	^10^
					1540 nm		1.0∙10^5a^	26^a^	
Silica fiber w/ WS_2_	Thermo-optic effect	No	Single mode	7.3 ms	980 nm	15 dB	2.1∙10^5a^	28.8^a^	^11^
Si_3_N_4_ waveguide	Thermo-optic effect	Yes	Single mode	5 µs	1550 nm	5.4 dB	50.4	10.1	^15^
Si_3_N_4_ waveguide	Kerr nonlinearity	Yes	Multi-mode	3.9 ps	1030 nm	2.2 dB	4.3	1.1∙10^6^	^9^
Si_3_N_4_ waveguide w/ graphene	Thermo-optic effect	Yes	Single mode	253.0 ns	1555 nm	10 dB	3.0	12	this work

^a^These devices include a fiber Mach-Zehnder interferometer (MZI) whose length is unknown. Here we use the fiber length covered by graphene or WS_2_ as the typical device length. But one should note that the actual device length should include the fiber length of MZI.

Our demonstrated device can be used as a switch for all-optical routing. For such an application, another straight waveguide should be added at the other side of the micro-ring resonator to form an “add-drop” configuration^1,20^. In this geometry, the control light and the incoming data stream are coupled to the first straight waveguide. The data stream can be routed either to the output port of the first waveguide (so called “through port”) or the output port of the second waveguide (so called “drop port”) by the presence or absence of a control light pulse.

## Conclusion

In conclusion, we have investigated all-optical control of light on a graphene-on-Si_3_N_4_ integrated chip using thermo-optic effect. The high thermal conductivity of graphene compensates the low thermo-optic effect in Si_3_N_4_ while maintaining the advantages brought by the insulator nature of Si_3_N_4_. In our experimental demonstration, the device has a switching time of 253 ns, a switching energy of ~50 nJ and a figure of merit of 3.0 nJ mm. The device has a good balance between power consumption and integration. Moreover, coupled-mode theory based simulation reveals the physical dynamics of the heating and cooling processes of the device. Our demonstration may pave the way for integrated all-optical signal processing in an insulator platform which allows large-scale integration with high power handling.

## Electronic supplementary material

Supplementary materials
