# Author Correction: All-optical control of light on a graphene-on-silicon nitride chip using thermo-optic effect

**DOI:** 10.1038/s41598-018-35544-8

**Published:** 2018-11-16

**Authors:** Ciyuan Qiu, Yuxing Yang, Chao Li, Yifang Wang, Kan Wu, Jianping Chen

**Affiliations:** State Key Laboratory of Advanced Optical Communication Systems and Networks, Department of Electronic Engineering, Shanghai Jiao Tong University, Shanghai, 200240 China

Correction to: *Scientific Reports* 10.1038/s41598-017-16989-9, published online 06 December 2017

The Acknowledgements section in this Article is incomplete.

“The work is partially supported by NSFC (No. 61505104, 61505105, 61178007, 51302285, 61235007, 61605112), the National Key R&D Program of China (No. 2016YFB0402501), the Science and Technology Commission of Shanghai Municipality (No. 15ZR1422800, 16XD1401400). Authors acknowledge the support of device fabrication by the Center for Advanced Electronic Materials and Devices of Shanghai Jiao Tong University”.

should read:

“The work is partially supported by NSFC (No. 61505104, 61505105, 61178007, 51302285, 61235007, 61605112), the National Key R&D Program of China (No. 2016YFB0402501), the Science and Technology Commission of Shanghai Municipality (No. 15ZR1422800, 16XD1401400) and Open Fund of IPOC (BUPT). Authors acknowledge the support of device fabrication by the Center for Advanced Electronic Materials and Devices of Shanghai Jiao Tong University”.

