# Supplementary material for: All-optical control of light on a graphene-on-silicon nitride chip using thermo-optic effect
Source: Sci Rep. 2017 Dec 6;7:17046. doi: 10.1038/s41598-017-16989-9 (PMC5719060; doi:10.1038/s41598-017-16989-9)
Supplement: Supplementary file 1 — Supplementary materials [file 41598_2017_16989_MOESM1_ESM.pdf]

All-optical control of light on a graphene-on-silicon nitride chip using thermo-optic effect: supplementary materials

Ciyuan Qiu, Yuxing Yang, Chao Li, Yifang Wang, Kan Wu,* and Jianping Chen

1. State Key Laboratory of Advanced Optical Communication Systems and Networks, Department of Electronic Engineering, Shanghai Jiao Tong University, Shanghai 200240, China

Correspondence and requests for materials should be addressed to K. W. (*kanwu@sjtu.edu.cn)

ABSTRACT

This document provides supplementary information to "All-optical control of light on a graphene-on-silicon nitride chip using thermo-optic effect". We discuss the parameter values in the coupled-mode theory (CMT) based simulation, the graphene induced waveguide propagation loss and the effect of different peak powers of the pump pulse.

1. CMT based simulation

In this section, we explain how the parameter values in Eq. (2-4) in the manuscript are determined and some supplementary fitting results for probe pulses. Based on the coupled-mode theory (CMT), the transmission of the resonator is given by [S1]

$$T = \left| \frac{j(\omega - \omega_0) + \tau_0^{-1} - \tau_e^{-1}}{j(\omega - \omega_0) + \tau_0^{-1} + \tau_e^{-1}} \right|^2 \quad \textbf{(S1)}$$

where ω is the frequency of the input light, ω_0 is the resonant frequency of the cold cavity. By fitting the measured transmission spectra of the device before and after the transfer of graphene, the values of τ_0 and τ_e can be obtained, as shown in Fig. S1. We obtain τ_e = 6.22 ps (unchanged), τ_0' =23.23 ps (before graphene transfer) and τ_0 =11.16 ps (after graphene transfer). And the coupling strength $|\kappa|^2$, expressed as $|\kappa|^2 = 2/\tau_e$ [S1], is equal to 3.21×10^{11} s^{-1}.

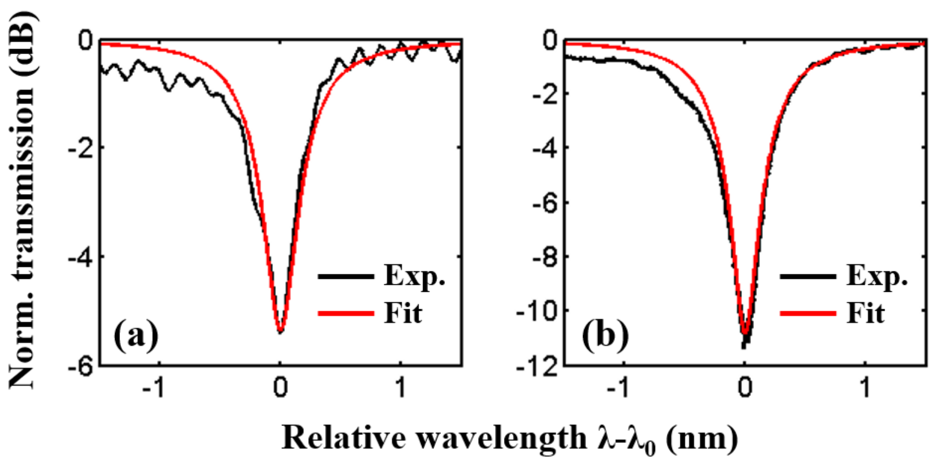

Fig. S1. Measured transmission spectra and fitting curves using CMT (a) before and (b) after graphene transfer.

Note that, $1/\tau_0'$ is the loss rate in the cavity due to the light scattering while $1/\tau_0$ is the loss rate in the cavity from both the light absorption and the light scattering. Thus the loss rate from the light absorption can be expressed as $1/\tau_{absorption} = 1/\tau_0 - 1/\tau_0'$. Furthermore, we define τ_{linear} as the power absorption rate in the resonator which is given by $\tau_{linear} = \tau_{absorption}/2 = 10.73$ ps.

By using the method illustrated in the manuscript, the output pulses with bias point B and C in Fig. 3(c) and 3(d) are also simulated, as shown in Fig. S2. The extinction ratio of the simulation results are slightly smaller than that of the experimental results, which may be due to the changed condition of optical coupling as explained in the manuscript.

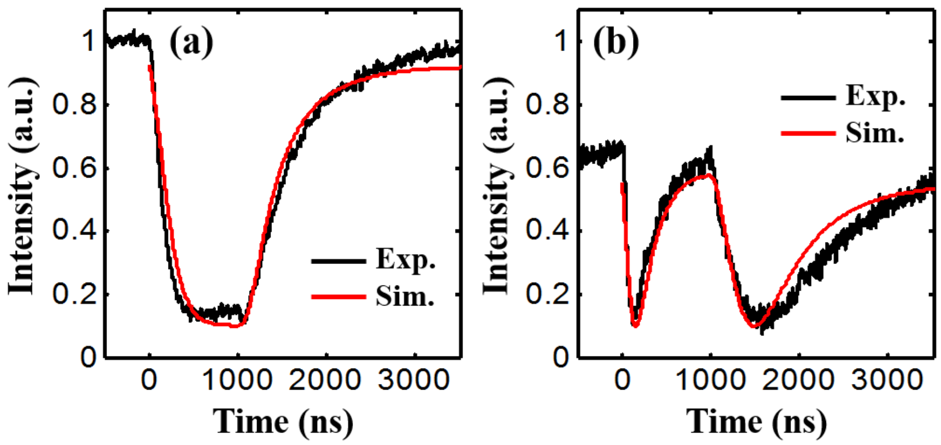

Fig. S2. Measured output pulses (black) and simulation results (red) with (a) bias point B and (b) bias point C.

2. Estimation of graphene absorption

The estimation of the graphene absorption is based on the theoretical model of a micro-ring resonator (MRR). The transmission of a MRR is given by [S2]

$$T = \left| \frac{t - t_r e^{j\varphi}}{1 - t t_r e^{j\varphi}} \right|^2 \quad \textbf{(S4)}$$

where t is the amplitude transmission of the coupler, t_r is the round-trip transmission of the MRR and φ is the round-trip phase change of the MRR. By fitting the transmission spectra before and after the transfer of graphene as shown in Fig. S3, one can obtain their values to be t=0.81 (coupler transmission, unchanged), t_r'= 0.942 (before graphene transfer) and t_r =0.89 (after graphene transfer). Therefore, the increased propagation loss of the graphene-Si_3N_4 waveguide is given by t_r/t_r'=0.945 or -0.49 dB. For a graphene length of 43.4 μm, the graphene induced waveguide propagation loss is 0.49 dB/43.4 μm=113 dB/cm.

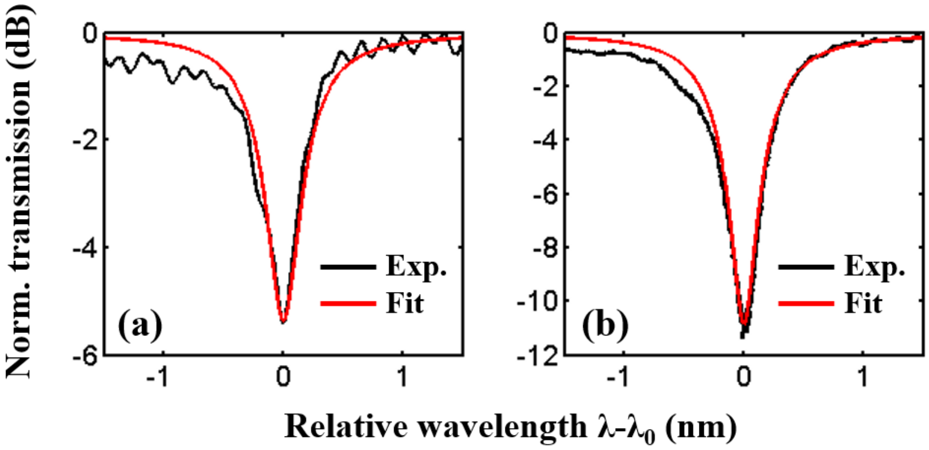

Fig. S3. Measured transmission spectra and fitting curves using MRR theoretical model (a) before and (b) after graphene transfer.

3. Effect of peak power of pump pulse

The peak power of the input pump pulse determines how fast the device is heated and thus the rising edge of the output probe pulse. To investigate the effect of peak power of the pump pulse, pump pulses with different peak powers and fixed pulse energy are applied. The average power is fixed to ~40 mW and the pulse period is fixed to 5 μs so that the total pulse energy is fixed to ~200 nJ. The duty cycle (denoted as δ) of the pulse train is tuned from 10% to 50%, so the peak power is equal to 40 mW/δ. The measured output probe pulses are shown in Fig. S4. It can be clearly found that the probe pulses generated by the pump pulses with a small duty cycle and high peak power have faster rising edges whereas the falling edges exhibit nearly the same decay rate. For the 10% duty-cycle case, the top of the probe pulse has not reached a steady state whereas for the 50% duty-cycle case, the probe pulse has reached a steady state but the peak intensity (or peak transmission) is only 86% of the probe pulse in the 20% duty-cycle case. The peak pump power, probe rise time and peak probe intensity are summarized in Table S2. In our experiments, a duty cycle of 20% is chosen to obtain a good balance between the rise time and the peak intensity (or peak transmission) of the probe pulses.

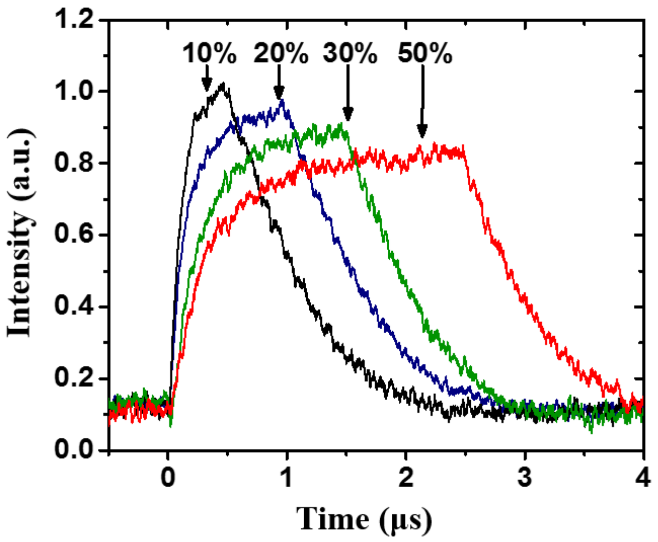

Fig. S4. Output probe pulses when the pump pulses with different duty cycles are applied.

Table S2 Properties of probe pulses generated by the pump pulses with different duty cycles

Duty cycle	Peak pump power (mW)	Probe rise time (ns)	Peak probe intensity (a.u.)
10%	400	126	1.02
20%	200	253	0.94
30%	133	354	0.86
50%	80	585	0.81

References

S1. C. Manolatou, M. Khan, S. Fan, P. R. Villeneuve, H. Haus, and J. Joannopoulos, IEEE J. Sel. Topics Quantum Electron 35, 1322 (1999).

S2. W. Bogaerts, P. De Heyn, T. Van Vaerenbergh, K. De Vos, S. Kumar Selvaraja, T. Claes, P. Dumon, P. Bienstman, D. Van Thourhout, and R. Baets, Laser Photonics Rev 6, 47 (2012).
